# Transcatheter Arterial Chemoembolization Is a Feasible Palliative Locoregional Therapy for Breast Cancer Liver Metastases

**DOI:** 10.1155/2010/251621

**Published:** 2010-10-28

**Authors:** Sung W. Cho, Krit Kitisin, David Buck, Jennifer Steel, Adam Brufsky, Roberta Gillespie, Allan Tsung, James W. Marsh, David A. Geller, T. Clark Gamblin

**Affiliations:** ^1^Division of Transplantation, Department of Surgery, University of Pittsburgh School of Medicine, UPMC Liver Cancer Center, Pittsburgh, PA 15213, USA; ^2^Division of Surgical Oncology, Department of Surgery, University of Pittsburgh School of Medicine, UPMC Liver Cancer Center, Pittsburgh, PA 15213, USA; ^3^Department of Radiology, University of Pittsburgh School of Medicine, UPMC Liver Cancer Center, Pittsburgh, PA 15213, USA; ^4^Department of Psychiatry, University of Pittsburgh School of Medicine, UPMC Liver Cancer Center, Pittsburgh, PA 15213, USA; ^5^Department of Medicine, University of Pittsburgh School of Medicine, UPMC Liver Cancer Center, Pittsburgh, PA 15213, USA; ^6^Division of Surgical Oncology, Medical College of Wisconsin, Milwaukee, WI 53226, USA

## Abstract

*Background*. Liver metastases are common in advanced breast cancer. We sought to evaluate the role of transcatheter arterial chemoembolization (TACE) in breast cancer patients with hepatic metastases. *Methods*. A retrospective review of ten patients with breast cancer who were treated with TACE for unresectable liver metastases (1998–2008). *Results*. All patients, median age 46.5, had received prior systemic chemotherapies. Adriamycin was administered for 6, cisplatin/gemcitabine for 2, cisplatin for one and oxaliplatin for one patient. Median number of TACE cycles was 4. Kaplan Meier survival analysis showed an increase in median survival for patients who responded to treatment when compared to those who did not respond (24 *vs* 7 months, *P* = .02). *Conclusions*. This is one of the largest series of breast cancer patients with liver metastases treated with TACE. It suggests that TACE is a feasible palliative option and warrants further investigations.

## 1. Introduction

Liver metastases are found in approximately 50% of patients with breast cancer during their lifetime [1]. In general, such liver involvement indicates a disseminated disease and portends a poor prognosis with median survival of approximately 4 months [2–4]. However, the liver is the only site of metastasis in 5 to 25% of breast cancer patients [3, 5]. Liver-directed therapy may delay progression of hepatic disease and minimally impact a patient's quality of life. Until recently, treatment of liver metastases has been limited to systemic agents such as chemotherapy, hormonal therapy, and biological therapy. In carefully selected patients, however, locoregional therapy to control hepatic tumor burden has also been attempted by means of resection, radio frequency ablation or hepatic artery therapy [6–13]. 

Transcatheter arterial chemoembolization (TACE) is a regional therapy that can be offered to patients with unresectable liver metastases. It delivers a high dose of chemotherapy directly to the liver metastasis in an attempt to minimize systemic side effects. The chemotherapeutic effect of TACE on tumor cells is augmented by the embolization-induced tumor ischemia of the therapy [14]. It has a proven beneficial role in the treatment of hepatocellular carcinoma as well as hepatic metastases from colorectal cancer and neuroendocrine tumors [15–17]. However, there is currently a paucity of data regarding its role in the management of metastatic breast cancer. We sought to evaluate efficacy and morbidity of TACE in selected patients with unresectable liver metastases from breast cancer.

## 2. Materials and Methods

Approval from the Institutional Review Board was obtained for a retrospective review of patients treated between January 1998 and January 2008. Ten patients with a diagnosis of breast cancer and unresectable liver metastases who were treated with TACE were identified during this period. The main inclusion criteria for TACE were liver-dominant tumor burden from breast cancer as seen on CT scan, progression on systemic chemotherapy, and good performance status. Patient with limited and indolent extrahepatic disease, which represented less than 10% of the disease burden and with the disease not progressing, was included in the study. Patients with evidence of hepatic failure such as total bilirubin greater than 3.0 mg/dL, ascites, or encephalopathy were excluded from TACE. Patient demographics, radiological and pathologic data on the primary and metastatic tumors, details regarding use of systemic chemotherapy, use of hormonal and biological treatments, and choice of TACE agents were sought. Morbidity of TACE procedures was examined from documentary and laboratory evidence. Clinical notes and electronic patient records were used to obtain follow-up and survival information. 

### 2.1. TACE Protocol

Prior to the start of the first cycle of TACE, a CT scan of the chest, abdomen and pelvis was performed to assess baseline tumor burden. A tissue biopsy of the hepatic lesions was obtained to confirm the origin of breast cancer, and basic laboratory values such as total bilirubin, albumin, and prothrombin time were obtained to assess baseline liver function. 

**Figure 1:**
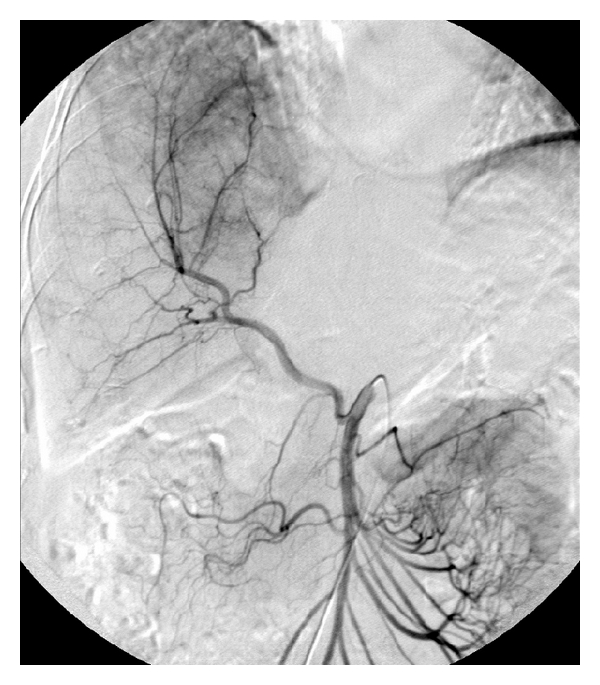
A selective angiogram of the superior mesenteric artery reveals the replaced right hepatic artery. Tumor-associated hypervascularity in the liver is demonstrated.

All the procedures were performed under a well-established protocol. In the interventional radiology suite under local anesthesia and intravenous sedation, the right common femoral artery was accessed. Using a standard 5 French curved catheter, an angiogram was performed to assess hepatic vascular anatomy and to examine tumor-associated vascularity (Figure 1). Routine use of triphasic intravenous contrast CT scan of liver and angiography was performed to define any anatomic vascular variants such as accessory or replaced hepatic vessels; and these variants were included in the treatment plan on diagnosis. Selective catheterization of hepatic artery branches was then performed in a coaxial manner using a 3 French microcatheter to identify tumor blush. After confirming appropriate microcatheter position, infusion of chemotherapy (adriamycin in 6 patients, cisplatin and gemcitabine in 2 patients, cisplatin in one patient, and oxaliplatin in one patient) was completed. For each TACE cycle, the dose of adriamycin ranged from 40 to 80 mg/m^2^, cisplatin 125 to 165 mg/m^2^, gemcitabine 1250 to 1500 mg/m^2^, and oxaliplatin 85 mg/m^2^. The choice of the chemotherapeutic agent depended on prior chemotherapy regimens. Chemotherapy was first administered to the hepatic lobe that contained the dominant tumor burden. In cases of bilobar involvement, each hepatic lobe was treated separately in different TACE cycles. Following arterial chemoinfusion, embolization of the selected arterial branch was performed to near stasis of antegrade flow. Embolization was achieved using Biospheres (Biosphere Medical, MA, USA), Gelfoam (Pfizer Inc, NY, USA), or Contour (Boston Scientific, MA, USA). All patients were observed overnight following the procedure, and laboratory tests were performed to assess side effects such as bone marrow suppression or liver dysfunction.

### 2.2. Assessment of Tumor Response

Six to eight weeks following each TACE cycle, a contrast-enhanced CT scan of the chest, abdomen and pelvis was obtained to assess intrahepatic tumor response to previous TACE and extrahepatic disease. CT scans detailing the size and the number of liver metastases were used to define radiological tumor response to the previous TACE session. RECIST criteria were used to grade the CT scan findings as progressive disease, stable disease, partial response, or complete response [18]. Assessment of radiological tumor response was carried out retrospectively for each patient at two time points, after the third TACE and after the last TACE. TACE cycles were continued while response was observed or until radiographic progression was documented.

### 2.3. Data Analysis

Data were entered and verified in SPSS (version 14, Chicago, IL). Descriptive statistics were performed to provide information on demographic and disease specific variables. Kaplan-Meier survival analysis was carried out to test the differences in survival after the start of TACE therapy between TACE responders (stable disease, partial or complete response) and nonresponders (progression of disease). 

## 3. Results

### 3.1. Patient and Tumor Characteristics

All patients were female, and the median age at the time of diagnosis of liver metastasis was 46.5 (range 29 to 60) (Table 1). Median time interval between diagnosis of primary breast cancer and of liver metastasis was 20 months (range 0 to 95). Primary tumor was treated with either lumpectomy with radiation (*n* = 4) or modified radical mastectomy (*n* = 5). One patient underwent an axillary lymph node dissection only for stage IV disease at presentation. Axillary lymph nodes were positive in all patients, and either estrogen or progesterone receptors were positive in six patients. 

Multiple bilobar hepatic metastases were found in nine patients and one large right lobe lesion was found in one patient. The range of the largest hepatic metastasis in each patient was 2.9 to 13.4 cm in largest dimension. The hepatic metastasis was deemed surgically unresectable in all patients. The liver was site of metastasis in only five patients, and the remaining five patients also had isolated bone metastasis. External beam radiation was used to treat symptomatic bone metastasis. 

All patients received systemic chemotherapy before the beginning of TACE therapy, and six patients underwent hormonal therapy (tamoxifen or aromatase inhibitors). Four patients also received trastuzumab. Systemic chemotherapy used for primary breast cancer included cyclophosphamide, methotrexate, 5-fluorouracil, adriamycin, cisplatin, taxol, and/or gemcitabine. Three patients received systemic chemotherapy for breast cancer with liver metastases at presentation. The remaining seven patients received second-line systemic chemotherapy such as taxol, navelbine, adriamycin, gemcitabine, and/or capecitabine for the liver metastases discovered subsequent to the diagnosis of primary breast cancer. Two patients also received autologous bone marrow transplantation after undergoing systemic chemotherapy for liver metastasis.

### 3.2. TACE Procedure

A total of 42 TACE sessions were performed (Table 2). The length of hospital stay was one day in all the sessions. Interval between TACE cycles was 6 to 8 weeks. Median number of TACE cycles administered was 4 (range 1 to 6).

### 3.3. Tumor Response and Morbidity

**Figure 2:**
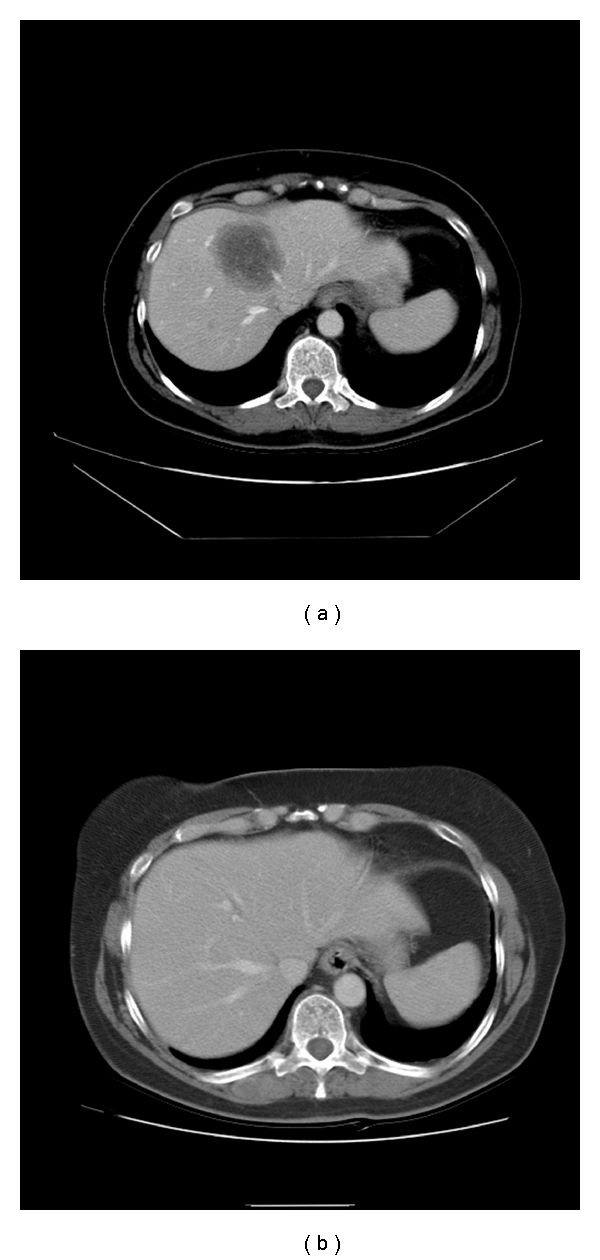
A computed topography of the abdomen shows a large hypodense hepatic metastasis before the TACE (a), which has disappeared following the TACE (b).

According to the RECIST criteria, surveillance CT scans showed that after the third cycle of the TACE, liver metastases progressed in 5 patients, stabilized in 3 patients, and partially responded in 1 patient. One patient received only one cycle of TACE. After the last cycle of the TACE, the disease progressed in 6 patients, stabilized in 2 patients, and partially responded in 2 patients (Figures 2(a) and 2(b)). Tumor markers (CA15.3, CA125 or CEA) decreased in 5 patients during TACE therapy. Of note, tumor markers decreased in all four patients who responded by radiological criteria following TACE. In contrast, five out of six patients who progressed on CT scan during TACE treatment also exhibited an increase in tumor markers. 

The most common side effect was postembolization syndrome (transient abdominal pain, nausea and/or vomiting) (*n* = 7). Three patients had transient neutropenia requiring treatment with filgrastim. Three patients had transient elevation of liver enzymes (ALT or AST) that did not require specific treatment.

### 3.4. Survival and Followup

**Figure 3:**
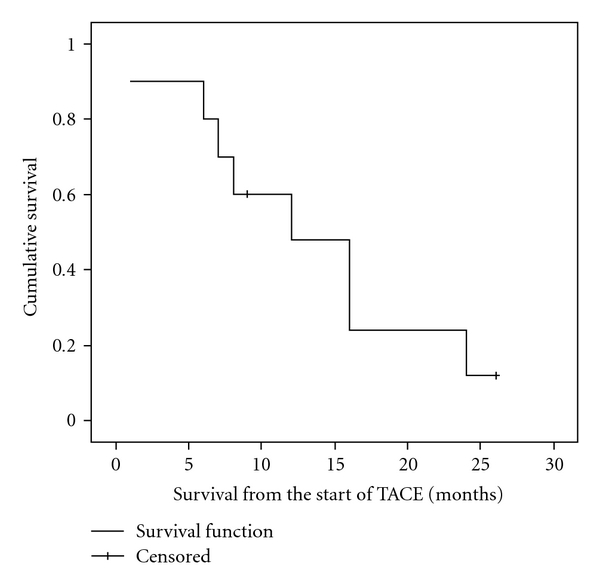
Kaplan-Meier survival curve of ten patients treated with TACE.

**Figure 4:**
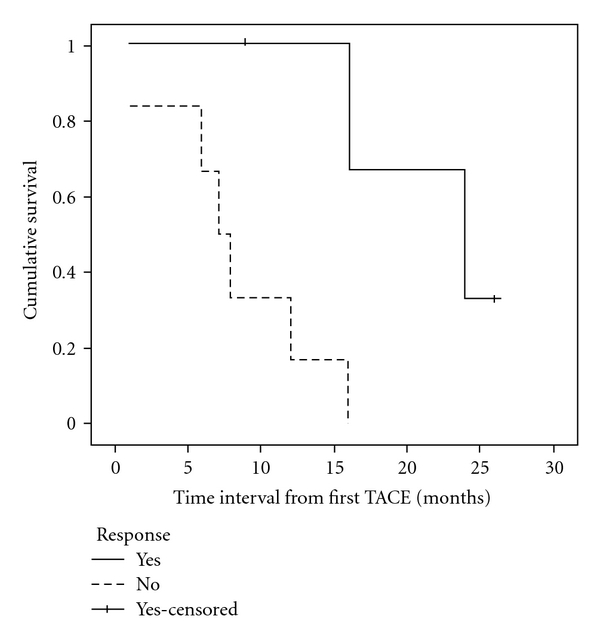
Kaplan-Meier survival curve of responders and non-responders to TACE therapy.

Median time intervals from the diagnosis of liver metastasis to death (*n* = 8) or last followup (*n* = 2) was 26 months (range 1 to 65). Six patients developed new or progressive extrahepatic metastasis during TACE therapy. Using Kaplan Meier survival analysis, median time interval from the first TACE therapy to death or last followup was 12 months (range 1 to 26, 95% confidence interval = 4.9–19.1 months) (Figure 3). Using radiological assessment of tumor response to TACE, a statistically significant increase in median survival was observed for four patients who responded to treatment (24 months) (partial response or stable disease) when compared to those six patients who did not respond to treatment (7 months) (progression of disease) (*P* = .02) (Figure 4). In addition, using Kaplan Meier survival analysis, comparing the three different types of chemoembolization, a statistically significant increase in median survival (16 months) was observed for six patients who received adriamycin when compared to four patients who received cisplatin and/or gemcitabine (12 months) or oxaliplatin (1 month) (*P* = .01).

## 4. Discussion

For patients with unresectable breast cancer liver metastases, the goal of treatment is to palliate symptoms and prolong survival without compromising the quality of remaining life. Recent advances in systemic therapies such as taxanes, aromatase inhibitors, and trastuzumab have helped to contain tumor progression in patients with advanced disease. Regional therapies such as hepatic resection have a role in selected patients, and retrospective case series have reported 5-year survival rates of 21% to 61% after resection of breast cancer liver metastases [6–9, 11, 12]. However, only a small number of patients are eligible for such a therapy, and there is therefore a need for an effective therapy that delays progression of unresectable but isolated liver metastases.

TACE can be performed with minimal disruption in patient's life. The length of hospital stay for all the TACE sessions in our study was only one day, and side effects observed after each cycle of TACE were transient and minor. Despite the ease of application and theoretical advantage of locoregional therapy in patients with isolated liver metastases, there have been only few reports of TACE in the setting of metastatic breast cancer. Our study reports ten patients treated with TACE after they had exhausted all the systemic therapeutic options available. Their extensive hepatic metastases excluded them from resection or radiofrequency ablation. We observed that by radiological and tumor marker criteria, 40% of hepatic metastases responded to TACE. Similarly, Li et al. reported that 35.7% of 28 breast cancer patients with liver metastases treated with TACE using fluorouracil, cisplatin, and doxorubicin responded on follow-up CT scans [19]. In another study, Giroux et al. showed that five out of eight metastatic breast cancer patients had a radiological response of hepatic metastases to cisplatin, doxorubicin, and mitomycin-based TACE [20]. Our series and previous published data therefore suggest that TACE may have a palliative role in delaying progression of isolated hepatic metastases. Of note, unlike two previous studies using triple chemotherapeutic agents, we observed that single-agent TACE in 8 patients and two agents in two patients stabilized or reduced the size of liver metastasis in 40% of patients, and adriamycin-based TACE conferred a statistically significant survival advantage (*P* = .01) when compared to cisplatin/gemcitabine or oxaliplatin-based TACE. This raises questions regarding the benefit of using multiple chemotherapeutic drugs for TACE. In support, Vogl et al. studied mitomycin-based single-agent TACE for 25 patients with liver metastases from breast cancer as a neoadjuvant therapy prior to laser-induced thermotherapy (LITT), and they observed a radiological response rate of 56% following TACE before LITT [21]. 

In addition, all four patients in our study who have responded to TACE had radiological evidence of response by the third TACE, and patients who did not show signs of response by the third cycle did not respond radiographically to further TACE. This suggests that patients who may benefit from continuing TACE therapy can be identified by measuring response during early cycles of TACE therapy. This selection process can be used to limit treatment among nonresponders. In addition, in our study, responses by radiological and tumor marker criteria were closely correlated, suggesting therapeutic efficacy of TACE on tumor burden in the liver. In this small series, such data should be considered pilot data, and thus a larger series investigation is needed.

In our study, TACE following systemic therapy for liver metastases was associated with median survival of 26 months from the time of diagnosis of liver metastasis, and at least 20% of patients survived 5 years. This finding is superior to that of the reported case series in which systemic therapy alone was used for liver metastases, which was associated with median survival of 4 to 10 months [3, 4]. Therefore, there may be a survival advantage for patients who also receive TACE in addition to systemic therapy for their hepatic metastases. In support, Schneebaum et al. also found that the median survival was 27 months for those patients who were treated with regional therapy (either resection or regional chemotherapy), which was significantly longer than the medial survival of five months for those patients who received systemic chemotherapy alone for liver metastases [22]. However, the median survival of 12 months from the start of TACE in our study indicates that TACE is clearly palliative and not a curative treatment. Six patients developed new extrahepatic metastases during TACE cycles, and four of them were bone metastasis. Radiation is an effective palliative treatment for symptomatic bone metastases, and studies have shown that bone metastases are not an influential factor on overall survival of patients with metastatic disease [23]. Interestingly, median survival from the start of TACE to death or last followup for the responders (*n* = 4) was 24 months compared to 7 months observed in non-responders (*n* = 6). Due to the small number of patients in our study, no firm conclusion can be drawn from this observation, but a statistically significant survival advantage was found for responders (*P* = .02). Our paper therefore provides preliminary data that locoregionally directed therapy to isolated liver metastases associated with radiological response can lead to prolonged survival. This finding warrants further investigations to validate the efficacy of TACE in this setting in a larger cohort of patients.

## 5. Conclusion

Here, we report a series of breast cancer patients with unresectable liver metastases who were treated with TACE. TACE was used in a patient population who had failed the first- and second- line systemic therapy, and it was associated with a median survival of 26 months from the diagnosis of liver metastases. This novel approach for breast cancer patients with liver metastases is an adjunct to existing systemic therapy, and it offers a well-tolerated palliative therapy in patients with historically dismal prognosis. In view of encouraging results from our study, further prospective randomized studies are warranted to assess TACE as a regional treatment for isolated hepatic metastases from breast cancer.

## Figures and Tables

**Table 1:** Patient characteristics of breast cancer primary, liver metastasis and details of prior systemic therapy.

Patient	Age	Treatment of Primary Ca	Axillary LN Status	Receptor Status	Extrahepatic metastasis before TACE	Interval between Diagnosis of Primary Breast Cancer and Liver Metastasis (in months)	Liver Metastasis Extent	Largest Liver Metastasis Size (in cm)	Previous Systemic Chemotherapy	Hormonal Treatment	Trastuzumab
1	35	mastectomy	positive	ER − PR − HER2/neu +	L4 bone	0	multiple bilobar	5.5 × 2.7	yes	no	yes

2	44	mastectomy	positive	ER + PR + HER2/neu +	T12 bone	95	multiple bilobar	12.2 × 5.2	yes	yes	yes

3	52	lumpectomy radiation, axillary LND	positive	ER − PR − HER2/neu −	None	14	multiple bilobar	13.4 × 8.2	yes	no	no

4	34	mastectomy	positive	ER + PR − HER2/neu −	None	24	multiple bilobar	6.9 × 5.2	yes	yes	no

5	40	lumpectomy radiation, axillary LND	positive	ER + PR +	multiple vertebral bones	60	multiple bilobar	10.0 × 9.0	yes	yes	no

6	60	mastectomy	positive	ER + PR +	None	0	multiple bilobar	13.0 × 8.6	yes	yes	no

7	51	axillary LND	positive	ER − PR − HER2/neu +	None	0	multiple bilobar	6.2 × 4.8	yes	no	yes

8	29	lumpectomy radiation	positive	ER − PR − HER2/neu +	None	16	multiple bilobar	4.9 × 4.1	yes	no	yes

9	49	lumpectomy radiation, axillary LND	positive	ER + PR + HER2/neu −	multiple vertebral bones, Calcaneus	93	multiple bilobar	2.9 × 2.1	yes	yes	no

10	54	mastectomy	positive	ER + PR − HER2/neu −	C3, C4, C5 vertebral bones	80	right lobe	11.0 × 8.0	yes	yes	no

**Table 2:** Details of TACE treatments and tumor response of breast cancer patients with hepatic metastases.

Patient	Chemothe-rapeutic Agent	Dose of chemotherapy used in each cycle (mg/m^2^)	Number of TACE cycles	Interval between first TACE and death/last F/U (in month)	Interval between diagnosis of liver metastasis and death/last F/U (in month)	Radiological response after third TACE	Radiological response after last TACE	Tumor marker response	New extrahepatic metastasis during TACE	Morbidity of TACE
1	adriamycin	80	3	6	12	progression	progression	CA125-increase	new left axillary LN	neutropenia

2	cisplatin/ Gemcitabine	125/1250	3	7	64	progression	progression	CA125-increase CA153-increase	new pulmonary metastasis	none

3	oxaliplatin	85	1	1	1	N/A	progression	CA153-increase CA125-decrease	none	nausea & vomiting

4	cisplatin	165	5	12	19	progression	progression	CA125-increase CA153-increase	new bony metastasis	nausea & vomiting

5	adriamycin	40	5	16	42	stable disease	partial response	CEA-decrease CA153-decrease	new bony metatasis	nausea, neutropenia

6	adriamycin	50~60	5	16	65	progression	progression	CA153-decrease	none	nausea, abdo pain neutropenia, anemia

7	cisplatin gemcitabine	150/1500	3	24	24	partial response	partial response	CA153-decrease	none	nausea

8	adriamycin	50	3	8	28	progression	progression	CA153-increase	none	abdo pain

9	adriamycin	50	4	9	9	stable disease	stable disease	CA153-decrease	new bony metastasis	abdo pain

10	adriamycin	40	10	26	39	stable disease	stable disease	CA153-decrease	new bony metastasis	none
